# Evaluation of antibody response to *Plasmodium falciparum *in children according to exposure of *Anopheles gambiae s.l *or *Anopheles funestus *vectors

**DOI:** 10.1186/1475-2875-6-117

**Published:** 2007-09-01

**Authors:** Jean Biram Sarr, Franck Remoue, Badara Samb, Ibrahima Dia, Sohibou Guindo, Cheikh Sow, Sophie Maiga, Seydou Tine, Cheikh Thiam, Anne-Marie Schacht, François Simondon, Lassana Konate, Gilles Riveau

**Affiliations:** 1 Association Espoir Pour La Santé (EPLS) BP 226 Saint-Louis, Senegal; 2 Unité UR 024 «Epidémiologie et Prévention» – Institut de Recherche pour le Développement (IRD), Campus IRD de Hann, Dakar, Senegal; 3 Laboratoire d'Ecologie vectorielle et Parasitaire, Département de Biologie Animale – Université Cheikh Anta Diop, Dakar, Senegal; 4 Laboratoire d'Entomologie Médicale – Institut Pasteur de Dakar, Senegal

## Abstract

**Background:**

In sub-Saharan areas, malaria transmission was mainly ensured by *Anopheles. gambiae *s.l. and *Anopheles. funestus *vectors. The immune response status to *Plasmodium falciparum *was evaluated in children living in two villages where malaria transmission was ensured by dissimilar species of *Anopheles *vectors (*An. funestus vs An. gambiae *s.l.).

**Methods:**

A multi-disciplinary study was performed in villages located in Northern Senegal. Two villages were selected: Mboula village where transmission is strictly ensured by *An. gambiae *s.l. and Gankette Balla village which is exposed to several *Anopheles *species but where *An. funestus *is the only infected vector found. In each village, a cohort of 150 children aged from one to nine years was followed during one year and IgG response directed to schizont extract was determined by ELISA.

**Results:**

Similar results of specific IgG responses according to age and *P. falciparum *infection were observed in both villages. Specific IgG response increased progressively from one-year to 5-year old children and then stayed high in children from five to nine years old. The children with *P. falciparum *infection had higher specific antibody responses compared to negative infection children, suggesting a strong relationship between production of specific antibodies and malaria transmission, rather than protective immunity. In contrast, higher variation of antibody levels according to malaria transmission periods were found in Mboula compared to Gankette Balla. In Mboula, the peak of malaria transmission was followed by a considerable increase in antibody levels, whereas low and constant anti-malaria IgG response was observed throughout the year in Gankette Balla.

**Conclusion:**

This study shows that the development of anti-malaria antibody response was profoundly different according to areas where malaria exposure is dependent with different *Anopheles *species. These results are discussed according to i) the use of immunological tool for the evaluation of malaria transmission and ii) the influence of *Anopheles *vectors species on the regulation of antibody responses to *P. falciparum*.

## Background

*Plasmodium falciparum *malaria is a major cause of human morbidity and mortality throughout tropical Africa. In sub-Saharan areas, malaria transmission is caused by several anopheles vectors, mostly *Anopheles gambiae *sensu stricto (s.s.) and *Anopheles arabiensis *from the *Anopheles. gambiae *complex, *Anopheles funestus *and *Anopheles pharaoensis *[1,2]. Depending on their bio-ecology, these species tend to alternate in different situations and seasons, since *An. funestus *breeds prolifically in swampy habitats with much vegetation, whereas freshwater members of the *An. gambiae *complex do best in small sunlit pools. The anthropophilic sibling species *An. arabiensis *and/or *An. gambiae *s.s. usually predominate in areas where the environmental conditions do not provide plentiful breeding sites for *An. funestus *[3], or where house-spraying has eliminated *An. funestus *[4]. Thus, *An. gambiae *sensu lato (s.l.) is the principal malaria vector in many epidemiological settings of the Afro-tropical region, such as Kenya [5], Tanzania [6], Zimbabwe [7], Zaire [8], and Senegal [9]. Nevertheless, in some local ecological environment (presence of permanent swamps and emergence vegetation), *An. funestus *can play a predominant role in malaria transmission. In Savannah areas, *An. funestus *has been shown to relay *An. gambiae *s.l., which reaches its peak of abundance in the early dry season [10].

In the Northern part of Senegal, malaria transmission is low, unstable and seasonal with an average of two to seven infective bites/person/year [11,12]. The management of Manantali and Diama dams, that have decreased the salinity gradient along the Senegal River, has probably contributed to the reappearance of *An. funestus *(which disappeared as a result of the drought in the 1970s) [12]. This situation has contributed to maintain malaria transmission at the beginning of the dry season [13]. The concomitant presence of *An. gambiae *and *An. funestus *vectors in this region provided an opportunity to survey this particular situation in which high risk of intense malaria transmission in populations presenting low anti-malaria immunity is commonly seen [14].

In many epidemiological studies, malaria transmission can be estimated by evaluating the density of *Anopheles *vectors infected by *Plasmodium *associated with the degree of infection/morbidity attributed to malaria in human [15]. Serological investigations have been also used to determine malaria transmission based on the antibody (Ab) levels against antigens to *P. falciparum *[16]. Recent immunological studies revealed that Ab directed to a panel of sporozoites and pre-erythrocytes antigens (NANP10, TRAP, SALSA, GLURP, STARP) or crude schizont extract increased with malaria exposure [17,18]; these Ab responses, therefore, estimate the level of malaria transmission rather than an immune-dependent protection [19].

To explore the risk and dynamics of malaria transmission, a longitudinal survey using an immunological marker, was conducted in Northern Senegal. Specific antibodies to *P. falciparum *blood stages have been screened using antigenic materiel derived from parasite lysates crude schizont [20] as a wide panel of parasite antigens [21]. This suggests that IgG Ab levels directed to wide antigens of *P. falciparum *represent a sort of global "picture" of anti-malaria immunity and gives information about malaria transmission.

In the present study, the objectives were

1) To evaluate IgG responses to *P. falciparum *wide antigens in two sites located in Northern Senegal (Mboula and Gankette Balla villages) where different *Anopheles *species are responsible for malaria transmission (*An. gambiae *s.l. and *An. funestus*, respectively)

2) To analyse the relationships between specific Ab levels and parasitological and entomological data

3) To analyse the one-year dynamics of Ab responses to *P. falciparum *in both villages according to the period of malaria transmission

## Methods

### Study population

The study was conducted in the north of Senegal, in two villages, Mboula and Gankette Balla, located along the Senegal River Basin, nearby Ferlo and the Lake Guiers, respectively. In this area, the prevalence and intensity of *P. falciparum *are known to be low in children less than 15 years of age [11]. This site is a dry savannah, with rainy season from July to October, and represents thus a typical area of the Sahelian and sub-Sahelian regions of Africa, with approximately 400 mm of rain by year. Malaria transmission in this area is seasonal from September to December [11,13].

The study population is in majority from the Wolof ethnic group. A longitudinal study was performed during one year, between June 2004 and June 2005 in both villages. Five passages (June, September, December 2004 and March, June 2005) were undertaken and, for each passage, a cohort of 150 children aged from one to nine years was selected for each village. The later passage included the distribution of permethrin-impregnated bet nets.

For each child, parasitological measurements of malaria were performed at each passage using thick blood smears (TBS) obtained by finger-prick. The smears were Giemsa-stained to identify *Plasmodium *species and the number of malaria parasites was counted. Parasite density was defined as the number of *P. falciparum *parasites/μl of blood. In the same way, capillary blood collection was done for each child at each passage for the determination of specific IgG levels by ELISA.

The present study followed ethical principles according to the Helsinki Declaration, and was approved by the Ethical Committee of the Ministry of Health of Senegal (CNRS; June 2004). Informed consent was obtained from the studied population.

### Entomological analysis

Adult mosquitoes were collected in June, September, December 2004 and March 2005 in both villages by human-landing collection. In each village, mosquito populations were caught in three selected households in six collection sites half indoor/half outdoor (7:00 p.m to 7:00 a.m) during two consecutive nights. The number of bites per human per night (BHN) was calculated by dividing the number of mosquitoes caught by the total person-night used for the period. Mosquitoes caught were brought to the laboratory, counted and identified morphologically to *Anopheles *species [22]. *Anopheles *infection rate was studied by ELISA (Enzyme-Linked ImmunoSorbent Assay) for *P. falciparum *circumsporozoite antigen (CSP). For all specimens, only 0.04% *An. funestus *collected in Gankette Balla was positive for *P. falciparum *CSP with an entomological inoculation rate (EIR) estimated to 3.00 infected bites. In contrast, transmission was not perceptible in Mboula whereas *An. gambiae *s.l. is the strict potential collected vector (EIR = 0).

### Evaluation of antibody response

ELISA was used to evaluate IgG directed to total extract of schizont. Total schizont antigen is a soluble extract of *P. falciparum *schizont lysate obtained from infected erythrocyte and kindly provided by D. Dive from the Institut Pasteur of Lille.

Schizont extract (7.5 μg/ml) were coated on flat-bottom microtiter plates (Nunc, Roskilde, Danemark) with 100 μL/well for 2 h 30 at 37°C. Plates wells were then blocked for 30 mn at room temperature with 200 μL of blocking buffer, pH 6.6 (Phosphate-Buffered Saline, PBS), 0.5% gelatin (Merck, Darmstadt, Germany) and washed one time with PBS, pH 7.2, 0.1% Tween 20 (Sigma Chemical Co). Individuals sera were incubated in duplicate at 4°C overnight at a 1/50 dilution (in PBS-Tween-0.1%). This dilution was determined as the optimum after several preliminary experiments. For detecting human IgG, plates were incubated for 90 min at 37°C with 100 μL of mouse biotinylated mAb to human IgG (BD Pharmingen, San Diego CA, USA) diluted 1/1000 in PBS-Tween 0.1%, after three times washing with PBS-Tween 0.1%. Plate wells were then washed four times with PBS-Tween and incubated for 30 minutes at room temperature with 100 μL of peroxydase-conjugated streptavidin (Amersham Biosciences, les Ulis, France). After washing six times with PBS-Tween, colorimetric development was carried out using ABTS (2.2'-azino-bis (3-ethylbenzthiazoline 6-sulfonic acid) diammonium; Sigma, St Louis, MO, USA) in 50 mM citrate buffer (Sigma, pH = 4, containing 0.003% H_2_O_2_) and absorbance (OD) was measured at 405 nm. Individuals results were expressed as ΔDO value calculated according to the formula: ΔDO = ODx-ODn, where ODx is the individual OD value of infected individuals and ODn was the individual OD value for each serum without antigen. The reproducibility of OD-positive values from IgG responders in the study children was verified in three later assays. A negative control (pool of sera from European individuals) was used for each assay. A subject was considered an immune responder if this ODx was higher than the ODn + (3 × SD) value.

### Statistical analysis

All data were analysed with Graph Pad Prism^® ^(Graph Pad, San Diego, USA) and R software version 2.3.1. After verifying that values in each group did not assume a Gaussian distribution, differences in Ab levels were tested by Mann-Whitney U-test and Kruskal-Wallis test between more than two groups. The non-parametric Friedman-test matched pair test was used to compare paired sera all along the follow up. Spearman's correlation was used to check for correlations between parameters. All differences were considered significant when P < 0.05.

## Results

### Evolution of specific IgG response during one year follow-up

The percentage of anti-malaria IgG responders, the *P. falciparum *prevalence in children and the intensity of exposure to both major *Anopheles *species bites (BHN) along the year follow-up, are presented on Table 1.

**Table 1:** Characteristics of studied populations during one year follow-up: *P. falciparum *prevalence, specific IgG immune responders and entomological data of *Anopheles *exposure.

	**Mboula (n = 62)**				**Gankette Balla (n = 89)**			
	*P.P.f^1^*	Responders^2 ^(%)	*An. gambiae^3 ^(BHN)*	*An. funestus^3 ^(BHN)*	*P.P.f^1^*	Responders^2 ^(%)	*An. Gambiae^3 ^(BHN)*	*An. funestus^3 ^(BHN)*
June 04	5.5	49/62 (79.03)	0.25	0	4	61/89 (68.54)	0	9.83
Sept 04	16.1	51/62 (82.26)	3	0	7.4	65/89 (73.03)	0.17	117
Dec 04	21.9	51/62 (82.26)	0.08	0	7	67/89 (75.08)	0.50	96.33
March 05	22	53/62 (85.48)	0	0	5.7	65/89 (73.03)	0.17	18.17
June 05	10	50/62 (80.65)	ND	ND	4.7	64/89 (71.91)	ND	ND

^*1*^*P.P.f*: Prevalence of *P. falciparum *in children (%).

^2 ^Number of responders in anti-schizonte IgG response (%).

^3 ^BHN = number of mosquito bites per human per night.

ND: Not Determined

The entomological data indicated that mainly *An. gambiae *s.l. was collected by using the human-landing method in Mboula village (only two *An. pharoensis *mosquitoes collected during the one year follow-up) suggesting that children were practically exposed only to bites by *An. gambiae *species. The exposure to *An. gambiae *bites was maximum in September but stayed low (BHN = 3). Whereas no infected *An. gambiae *was detected, moderate prevalence of *P. falciparum *was observed during the seasonal transmission (16 to 22%). Indeed, prevalence increased in September to reach a peak in December and March. It could be thus considered that *An. gambiae *species was the strict vector in Mboula. In contrast, in Gankette Balla, individuals were largely exposed to *An. funestus *bites as indicated by very high BHN compared to low *An. gambiae *BHN. In addition, exposure to *An. funestus *was higher in September and December and this species is the strict vector in this village. A very low prevalence of *P. falciparum *was observed during the year follow-up.

In both villages, high specific IgG responders were found with prevalence ranging from 68 to 85% (Table 1). For each village, the rate of responders was relatively constant during the year follow-up. A highest prevalence of *P. falciparum *infection was found in Mboula which presented differences according to the passage with a peak between December and March 2005 (Table 1). Therefore, the evolution of *P. falciparum *prevalence did not seem to influence the % of IgG responders in both villages.

**Figure 1:**
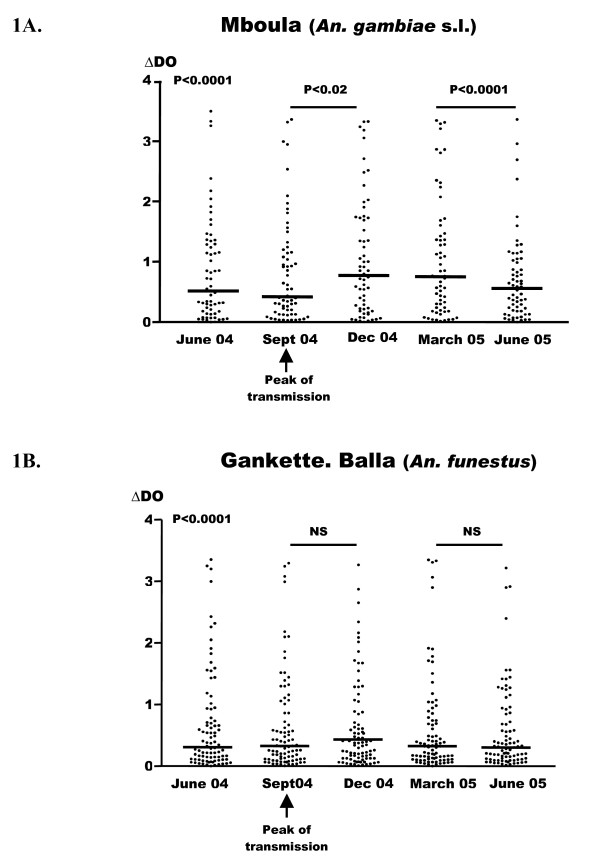
**Anti-*P. falciparum *IgG responses according to the malaria transmission periods in the two villages**. Individual absorbance (ΔDO) results obtained for each passage are shown only for children presents for all passages (n = 62/passage in Mboula and 89/passage in Gankette Balla). Figure 1C showed median values of antibody levels (expressed in median values) in children from Mboula and Gankette Balla. NS = No Significant.

In contrast to the percentage of responders, differences in the levels of specific antibody responses were observed between both villages (Figure 1). The highest Ab levels to schizont extract were observed in Mboula all along the studied period, compared to Gankette Balla (Figure 1C). In Mboula, a significant increase of specific IgG responses was observed between September to December (Figure 1A, P < 0.02). Thereafter, these responses mildly declined from March to June 2005 (Figure 1A, P < 0.0001). This variation of the specific IgG Ab levels according to months appeared roughly concomitant with the peak of malaria transmission (September to March – Table 1). In contrast, no variation of IgG Ab levels was observed along the year follow-up in Gankette Balla (Figure 1B).

### Specific IgG response and *P. falciparum *infection

**Figure 2:**
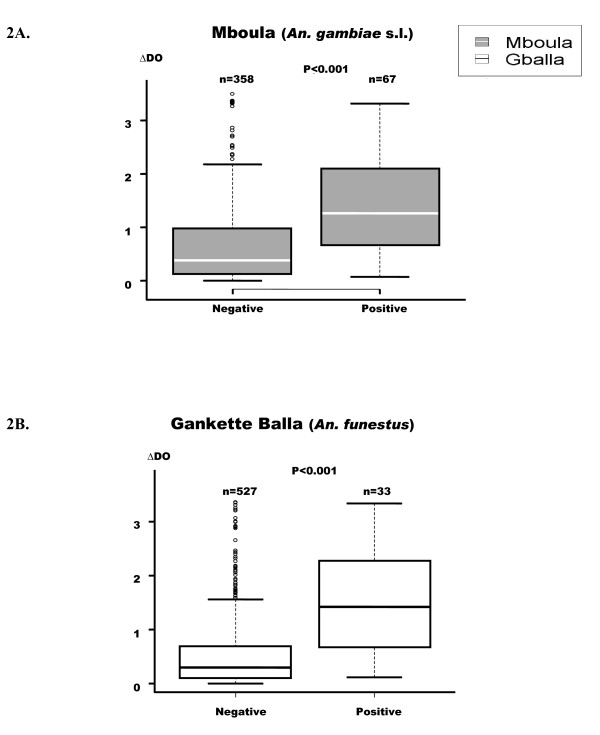
**Anti-*P. falciparum *IgG responses according to malaria positive or negative infection in both villages**. p value of the Mann-Whitney U-test, Box-whiskers plots illustrate medians with 25^th ^and 75^th ^percentiles, and whiskers for 10^th ^and 90^th ^percentiles. The upper whisker extends to the largest value below the 75th percentile plus the box height multiplied by 1.5. n = number of children with or without malaria positive infection.

IgG Ab levels directed to schizont extract were presented according to the presence or the absence of *P. falciparum *infection (Figure 2). Malaria infection was diagnosed by a positive thick blood smears in children. The presented results concern the cumulative data from all passages.

The children presenting malaria infection developed higher IgG response than negative children (P < 0.001) (Figures 2A and 2B). Similar results of IgG levels according to *P. falciparum *infection were found in both villages.

In addition, a correlation was observed between the intensity of malaria infection (number of *P. falciparum *parasite/μl of blood) and Ab responses in Mboula (r = 0.332, P = 0.02), whereas in Gankette Balla the correlation (r = 0.047, NS) was not significant.

### Specific IgG response and age

**Figure 3:**
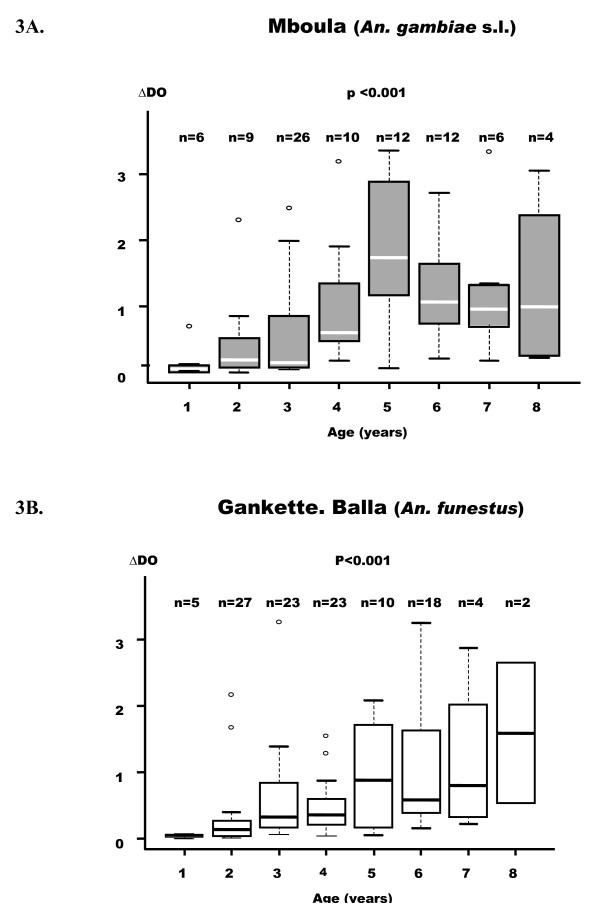
**Anti-*P. falciparum *IgG responses according to age in the two villages**. p value of the Kruskal-Wallis U-test. Box-whiskers plots illustrate medians with 25^th ^and 75^th ^percentiles, and whiskers for 10^th ^and 90^th ^percentiles. The upper or lower whisker extends to the largest value below the 75^th ^percentiles plus the box height multiplied by 1.5.

The levels of anti-shizont IgG after the period of transmission (December) according to the age of children was presented in Figure 3. The relationship between age and Ab levels appeared to be similar in both villages. Indeed, progressive increase of Ab levels was observed in children from one to five years of age and reached a peak at five. After this age, a slight decrease or a plateau was observed.

In Gankette Balla, specific IgG responses appeared to be correlated with age, even in children not infected by *P. falciparum *(r = 0.604, P = 0.004). This result suggests that the age-dependent increase of specific IgG response was not dependent to malaria status. In Mboula, this positive correlation was not significant in uninfected children (r = 0.195, P = 0.132).

### Comparison of specific IgG response with the intensity of exposure to *Anopheles*

**Figure 4:**
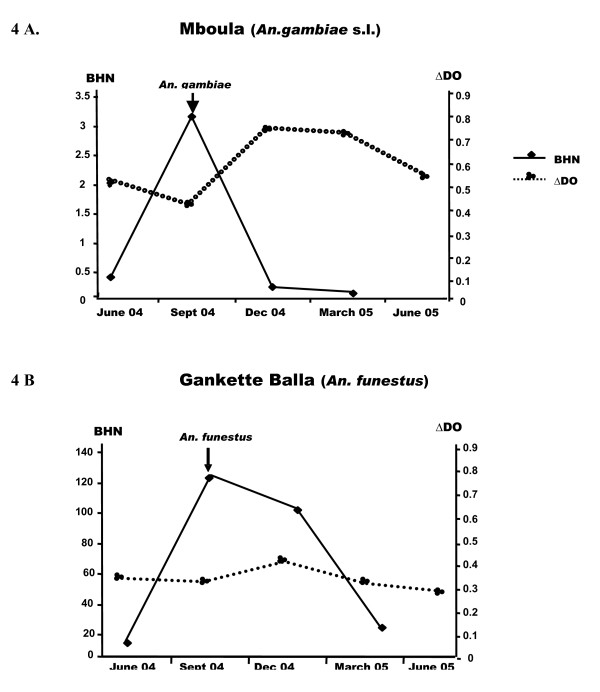
**Relationships between anti-*P. falciparum *IgG responses and the intensity of exposure to major *Anopheles *bites**. Right and left scale delineates respectively the number of bites per human per night (BHN) and *P. falciparum *IgG levels (ΔDO).

The relationship between specific IgG levels (median) and entomological data evaluating the intensity of exposure to both major *Anopheles *species (number of aggressive mosquitoes per person per night, BHN) were presented during one year follow-up (Figure 4).

In Mboula (Figure 4A), the exposure to *An. gambiae *increased during the period of high malaria transmission (September). This peak of *An. gambiae *BHN was followed by an increase in levels of specific IgG in December to March (Figure 4A). In contrast, in Gankette Balla, the peak of BHN in September was not followed by an evolution of IgG responses (Fig. 4B).

## Discussion

The main point of the study was the comparison of the specific Ab response in two villages where exposure to *Anopheles *bites and malaria transmission were caused by two different vector species (*An. gambiae *s.l. vs *An. funestus*). For this purpose, the evolution of anti-schizont IgG levels in relation to the intensity of exposure to *Anopheles *species was analysed during one year. The IgG response against whole *P. falciparum *extract antigens was thus assessed in children living on both villages according to intensity of exposure to *Anopheles *species, age, period of transmission and presence of malaria infection (Table 1, Figure 3, Figure 1 and Figure 2, respectively). Specific IgG responses differed between villages and passages and remained higher in Mboula. The peak of anti-malaria IgG response appeared to follow the increase of prevalence observed in September in Mboula. In contrast, no season effect was observed in Gankette Balla. The high rate of malaria infection observed in Mboula during seasonal transmission and the very low and continuous prevalence in Gankette Balla could be one explanation of these differences observed between both villages. This positive correlation between high or low prevalence of *P. falciparum *infection and Ab responses levels has been reported in other epidemiological studies [23,24].

In spite of the differences of specific IgG responses between villages, positive association of immune response with *P. falciparum *infection was observed. This association was independent of vector species (similar in both villages) and could result from a booster effect of the presence of long-lasting presence of the parasite on the specific immunity [25,26]. The studied specific Ab responses appeared thus to be associated with malaria infection rather than anti-malarial protective immunity. The association observed between malaria infection and anti-*P. falciparum *IgG responses in both villages need the use of better immunological tools in order to closely evaluate the intensity of transmission. For this purpose, it has been shown that the Ab responses against different sporozoite (TRAP, CSP, NANP10) or pre-erythrocyte antigens (LSA, STARP, SALSA) are positive serological markers of malaria transmission [18,19,27]. This research is currently on the way for both studied villages in order to evaluate which adequate antigens could be used as transmission marker in children exposed to malaria, even if transmission is caused by different vector species. In addition, IgG isotypes response could be investigated as another tool to evaluate malaria transmission in a more precise manner. Indeed, Ramasamy *et al *[28] reported a strong relationship between cytophilic IgG1 and IgG3 Ab responses directed to sporozoite and merozoite surface antigens and malaria transmission dynamism. In addition, non-cytophilic IgG2 and IgG4 response could be, an indicator of primo-invasion [29,30].

It has been also reported that specific IgG responses increased progressively in children aged from one- to five years and then stay high until eight. This influence of age was similar in both villages and appeared to be independent to of malaria status. This intrinsic increase of anti-malaria Ab response with age was commonly reported in children living in malaria areas and in populations highly exposed to infection [31,32]. This age-dependent immune response was thought to correspond to naturally acquired immunity and to long-term memory cells of the antigenic repertoire to *P. falciparum *[25]. In the present study, the fact that infected children were exposed to different *Anopheles *species and that the prevalence of *P. falciparum *infection was very different between villages, did not appear to be a factor of variation of the age-dependent development of anti-malarial IgG responses.

In Mboula, the peak of *An. gambiae *s.l. exposure (September) was followed by the high IgG responses in December, which remained stable until March. In contrast, no difference of Ab responses was observed in Gankette Balla according to season, whereas the exposure of *An. funestus *vector was very high in September-December. This situation of high exposure to *An. funestus *species accompanied with a very low prevalence of *P. falciparum *infection was rarely observed in the epidemiological studies which have taken place in Senegal and elsewhere in Africa [33,34]. Several extrinsic and intrinsic epidemiological factors (genetic background of individuals, different history of malaria infection and use of anti-malaria prevention...) could be involved to explain these profound differences between the development of anti-malarial immunity in both villages exposed to two separated *Anopheles *vectors. Nevertheless, these immune differences could be attributed to the characteristics of malaria transmission in the areas studied. In Gankette Balla, high exposure to larval *An. funestus *species, resulting in the reconstitution of their breeding sites, could provide optimal conditions to aquatic stages and thus allow the emergence of adults. *An. funestus *collected in Gankette Balla were young, the most part having never laid and strikingly zoophilic; they seemed to present a very low vectorial capacity (Dia, unpublished). This particular situation could explain the low malaria transmission and low prevalence of *P. falciparum *observed in this village and could clarify the absence of seasonal variations of anti-malarial Ab response. In contrast, the low, but marked, season-dependent exposure to *An. gambiae *s.l. vector in Mboula appeared to induce a significant increase of specific IgG response. The bionomics of *An. gambiae *s.l. is closely related to human populations (anthropophilic and endophilic) and its high susceptibility to human malaria parasite endows this vector with a high vectorial ability [35]. This is why, there are significant behavioural differences between both species with a higher anthropophilic rate (75.82%) of *An. gambiae *s.l. in Mboula compared to *An. funestus *(33.18%) in Gankette Balla (Dia, unpublished).

The present study shows a close association between the presence of *P. falciparum *infection and the level of anti-schizont IgG response. However, it is conceivable that the bite by different *Anopheles *species vector which caused transmission could influence the anti-malarial Ab responses. Indeed, it is now well known that the saliva of arthropod vectors presents an immunomodulatory effect [36-38]. In addition, it has been suggested that mosquito saliva could influence parasitaemia by regulating anti-*P. falciparum *protective immunity [39,40]. In the present study, it has been shown that the profile of some salivary proteins and their respective immunogenicity could be different according to *An. funestus *and *An. gambiae *species (Remoue, personal communication). The identification of the different proteins between both species is actually under investigation. The variation of salivary proteins expression according to mosquito species and their secretion during the bite has been also observed between *Aedes *species [41]. It could thus be possible that the salivary proteins of different *Anopheles *species could show dissimilarity and induces a different immune regulation on the development of anti-malarial immunity. Recently, it has been demonstrated that IgG responses to *Anopheles *saliva is detected in children living in malaria area and represent a marker of the intensity of *An. gambiae *exposure [42]. The comparison of specific anti-*P. falciparum *and anti-saliva Ab responses in these villages according to the season is currently under investigation in the same cohort. This project will be one step to validate the hypothesis that the bite by different *Anopheles *vectors could influence the anti-parasite immune response.

## Conclusion

The main original results of the present study show that the acquired anti-*P. falciparum *IgG response in children was different in two areas where malaria transmission was ensured by different *Anopheles *species. Previously, it has been demonstrated that in a *Leishmania *animal model, the immune regulation of *Phlebotomus *saliva can induce a substantial effect on the development of infection and pathology [43,44]. Thus, the present study suggests that the development of anti-parasite immune response could be influenced by the vector bite. The multi-disciplinary approach of the malaria transmission, bringing entomological, parasitological, epidemiological and immunological data, appeared to be necessary to validate this hypothesis.

## Authors' contributions

JBS and FR have equally contributed to the design, acquisition, analysis, interpretation of data and manuscript drafting.

ID, LK contributed to conception of study and contributed markedly to the analysis of entomological data.

SG, CS, SM, ST, CT, for field activities and microscopic examinations.

AMS provided expertise and technical support for evaluation of the immune responses.

FS participated in the conception and coordination of the study and helped to draft the manuscript.

GR provided the scientific supervision in Saint Louis and revised the manuscript.

This study was performed in the framework of the "Pal-Fleuve programme" which is a structure developing multidisciplinary approaches (entomology, parasitology, immunology...) and complementary researches on malaria exposure/transmission in Northern Senegal. This programme includes four units of research: EPLS (Association Espoir Pour La Santé) – Saint Louis; IRD-UR024 (Epidémiologie et Prévention) – Dakar; UCAD (Université Cheikh Anta Diop) – Dakar and IPD (Institut Pasteur de Dakar).
